# Extending the Depth of Focus of an Infrared Microscope Using a Binary Axicon Fabricated on Barium Fluoride

**DOI:** 10.3390/mi15040537

**Published:** 2024-04-17

**Authors:** Molong Han, Daniel Smith, Tauno Kahro, Dominyka Stonytė, Aarne Kasikov, Darius Gailevičius, Vipin Tiwari, Agnes Pristy Ignatius Xavier, Shivasubramanian Gopinath, Soon Hock Ng, Aravind Simon John Francis Rajeswary, Aile Tamm, Kaupo Kukli, Keith Bambery, Jitraporn Vongsvivut, Saulius Juodkazis, Vijayakumar Anand

**Affiliations:** 1 Optical Sciences Centre and ARC Training Centre in Surface Engineering for Advanced Materials (SEAM), School of Science, Computing and Engineering Technologies, Swinburne University of Technology, Hawthorn, VIC 3122, Australia; molonghan@swin.edu.au (M.H.); danielsmith@swin.edu.au (D.S.); soonhockng@swin.edu.au (S.H.N.); sjuodkazis@swin.edu.au (S.J.); 2 Institute of Physics, University of Tartu, 50411 Tartu, Estonia; tauno.kahro@ut.ee (T.K.); aarne.kasikov@ut.ee (A.K.); vipin.tiwari@ut.ee (V.T.); agnes.pristy.ignatius.xavier@ut.ee (A.P.I.X.); shivasubramanian.gopinath@ut.ee (S.G.); aravind@ut.ee (A.S.J.F.R.); aile.tamm@hm.ee (A.T.); kaupo.kukli@ut.ee (K.K.); 3 Laser Research Center, Physics Faculty, Vilnius University, Sauletekio Ave. 10, 10223 Vilnius, Lithuania; dominyka.stonyte@ff.vu.lt (D.S.); darius.gailevicius@ff.vu.lt (D.G.); 4 School of Electrical and Computer Engineering, Ben Gurion University of the Negev, P.O. Box 653, Beer-Sheva 8410501, Israel; 5 Infrared Microspectroscopy (IRM) Beamline, ANSTO—Australian Synchrotron, Clayton, VIC 3168, Australiajitrapov@ansto.gov.au (J.V.); 6 Tokyo Tech World Research Hub Initiative (WRHI), School of Materials and Chemical Technology, Tokyo Institute of Technology, 2-12-1, Ookayama, Meguro-ku, Tokyo 152-8550, Japan

**Keywords:** infrared microscopy, axial resolution, binary axicon, photolithography, femtosecond ablation, chemical imaging

## Abstract

Axial resolution is one of the most important characteristics of a microscope. In all microscopes, a high axial resolution is desired in order to discriminate information efficiently along the longitudinal direction. However, when studying thick samples that do not contain laterally overlapping information, a low axial resolution is desirable, as information from multiple planes can be recorded simultaneously from a single camera shot instead of plane-by-plane mechanical refocusing. In this study, we increased the focal depth of an infrared microscope non-invasively by introducing a binary axicon fabricated on a barium fluoride substrate close to the sample. Preliminary results of imaging the thick and sparse silk fibers showed an improved focal depth with a slight decrease in lateral resolution and an increase in background noise.

## 1. Introduction

Axial resolving power (ARP), an ability to discriminate information along the longitudinal direction, is an important characteristic of all imaging systems [1]. ARP is dependent upon the numerical aperture (NA) and the wavelength λ, given as ~λ/NA^2^. As the lateral resolving power (LRP) - the ability to discriminate information along the transverse direction is given as ~λ/NA, any attempt to change the ARP by changing NA or λ also changes the LRP. While a high LRP–high ARP pair is desirable for most experiments, there are also special cases and scenarios where a high LRP–low ARP pair is desirable. In the case of sparse, thick samples (such as a bunch of fibers and fluorescent samples), the information in one axial plane does not overlap with information in another axial plane laterally. Therefore, if the ARP can be decreased without affecting the LRP, then information from multiple planes can be imaged in a single camera shot [2]. This pair (high LRP–low ARP) can enable rapid imaging of thick and sparse samples.

One imaging solution to achieve the above condition is to implement digital holography techniques [3]. In the digital holography technique, only a few camera shots are recorded, and the information from multiple planes are reconstructed by numerical back propagation of the recorded holograms, or in simple words digital refocusing. Certain digital holography techniques, such as Fresnel incoherent correlation holography (FINCH), have a naturally surprising LRP–ARP pair [4,5,6,7], where their ARP and LRP are lower and higher, respectively, than those of conventional imaging systems with the same NA. However, digital holography systems such as FINCH are often difficult to implement, as they require many optical components and active devices such as spatial light modulators (SLMs) and, in most cases, require changes in the optical configuration itself. Consequently, it is difficult to implement digital holography techniques such as FINCH in commonly available commercial microscopes.

Another solution to the above problem is to record the microscope’s 3D point spread function and use deconvolution methods to reconstruct the object information [8,9,10]. While deconvolution methods are valuable, the reconstructed images often suffer from reconstruction errors and artifacts. While deep learning methods are now developed to improve the performances of holography systems, especially in the reconstruction part, there are still challenges present that include obtaining a large data set and time-consuming training processes [11,12]. Computational imaging methods have been developed to tune ARP independent of LRP but require special optical configurations (SLMs) and, therefore, cannot be used with existing commercial microscopes [13,14,15,16,17].

A well-known, tested solution to achieve the unusual state of high LRP and low ARP is by using an axicon instead of a lens for imaging [18,19,20,21,22]. Axicons generate non-diffracting Bessel beams and, therefore, have a long focal depth when used for imaging applications [23,24,25,26,27,28,29,30,31,32]. In many studies on extending the depth of field using an axicon, the optical configuration has been modified by replacing the lens with an axicon [33] and attaching an axicon to a lens [34,35,36], and, in some cases, the Bessel beam generated from the axicon was used for illumination of the sample [37,38]. The above modifications are often challenging to implement in an existing commercial microscope. In some cases that use a visible light illumination, a solid axicon may be attached. Still, it may be complicated and impossible in the case of other wavelengths such as infrared (IR), X-rays, and Gamma rays.

One special case is discussed next. Most synchrotron facilities tend to be unique in their capabilities and feature finite access times for various users. In this particular case, the Australian Synchrotron has a state-of-the-art IR microspectroscopy system with two illumination sources, namely the Globar™ IR source and a synchrotron IR beam. The microspectroscopy system with the synchrotron IR beam is essentially the most used system for high-resolution chemical imaging of a diverse range of samples from many research disciplines, because the high-demand beamtime grant is limited to a maximum of approximately 1600 h tri-annually. The IR imaging study of thick samples layer-by-layer is a valuable yet time-consuming procedure that is either overlooked or skipped due to the limitation of the access and period of available beamtimes. The low ARP–high LRP pair can enable a rapid and routine analysis for a wide range of thick and sparse samples at the Australian synchrotron.

In this study, we propose and demonstrate an improved optical configuration that allows introducing an axicon close to the object, between the object and the imaging lens for extending the focal depth of an IR microscope. In particular, the initial testing was performed at the Australian Synchrotron facility using a highly intense, highly collimated synchrotron IR beam. The full experiment was carried out in an IR microscope with a Globar™ IR light source at the Australian Synchrotron. Supporting experiments with solid axicon and diffractive masks on an SLM have been demonstrated in the visible region. The above-mentioned experiments in different configurations and wavelengths are added to demonstrate the potential and broader applicability of the developed method.

This manuscript consists of nine sections. In the next section, the methodology of extending the depth of focus using an axicon with the object–axicon close to each other is presented. The simulation results of the system are presented in the third section. Optical experiments using a refractive lens and axicon are presented in the fourth section. Optical experiments with a modified configuration using an SLM is presented in the fifth section. The fabrication methods and results are discussed in the sixth section. The initial testing results with synchrotron near IR (NIR) beam are presented in the seventh section. The results of the final experiments using an internal Globar™ IR light source in a commercial IR microscope are presented in the eighth section. The conclusion and future perspectives are presented in the final section.

## 2. Methods

**Figure 1:**
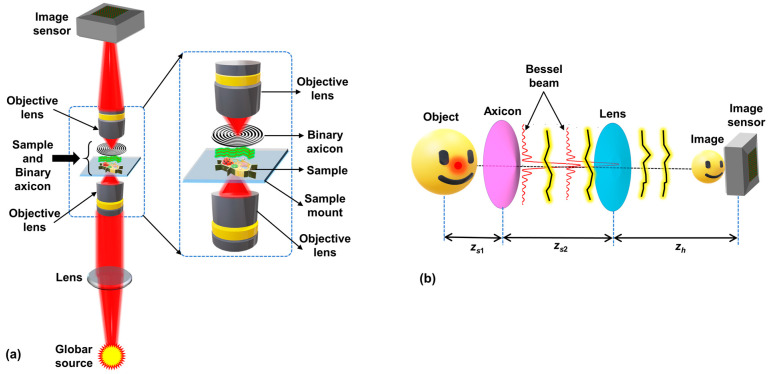
(**a**) Optical configuration of microscope with object and axicon close to each other. (**b**) Simplified optical configuration for analysis. The distances between the object and axicon, axicon and lens, and lens and image sensor are *z_s_*_1_, *z_s_*_2_, and *z_h_*, respectively.

A simplified optical configuration of a typical microscope with the axicon and object close to each other is shown in Figure 1a. A simplified optical configuration is shown in Figure 1b for theoretical analysis. In this study, only spatially incoherent illumination is considered, as the IR microscope discussed in the previous section has such an illumination source. As in any incoherent imaging system, the theoretical calculation is performed for an object point and directly extended to a complicated object. The light from the Globar^TM^ source is collimated by a refractive lens. The collimated light is collected by an objective lens and focused on the sample plane, as shown in Figure 1a. The light from the object in the sample plane is incident on a binary axicon placed close to the sample and collected by a second objective lens. This second objective lens forms the image of the object on the image sensor. The magnified view of two objective lenses, the sample and the binary axicon, is shown in Figure 1a. The simplified configuration created for theoretical and simulation studies shown in Figure 1b begins from the object and ends with the sensor. Just like the original configuration shown in Figure 1a, an axicon is present after the object but at a closer distance with a lens in between the axicon and the image sensor. Let us consider an object point emitting light with an amplitude of $\sqrt{I_{s}}$. The light from this object point is incident on a thin axicon located at a distance of *z_s_*_1_, with a phase function given as $\exp\left(-i2\pi \Lambda^{-1}R\right)$, where Λ is the period of the modulo-2π structure and $R={\left(x^{2}+y^{2}\right)}^{1/2}$. The complex amplitude after the axicon is given as $\sqrt{I_{s}}C_{1}L\left(\frac{{\bar{r}}_{s}}{z_{s1}}\right)Q\left(\frac{1}{z_{s1}}\right)\exp\left(-i2\pi \Lambda^{-1}R\right)$, where $Q(b)=\exp\left(i\pi b\lambda^{-1}R^{2}\right)$ is a quadratic phase function, $L\left(\frac{\bar{s}}{z_{s1}}\right)=\exp\left[i2\pi {\left(\lambda z_{s1}\right)}^{-1}\left(s_{x}x+s_{y}y\right)\right]$ is a linear phase function, and *C*_1_ is a complex constant. In general, when a plane wave is incident on the thin axicon, a Bessel beam *J*_0_ is generated. The complex amplitude reaching a refractive lens with a phase of $\exp\left[-i\pi ({\lambda f)}^{-1}R^{2}\right]$ located at a distance of *z_s_*_2_ from the axicon is given as $\sqrt{I_{s}}C_{1}L\left(\frac{{\bar{r}}_{s}}{z_{s1}}\right)Q\left(\frac{1}{z_{s1}}\right)\exp\left(-i2\pi \Lambda^{-1}R\right)⨂Q\left(\frac{1}{z_{s2}}\right)$, where ‘⨂’ is a 2D convolutional operator. The complex amplitude after the lens is given as $\left\{\sqrt{I_{s}}C_{1}L\left(\frac{{\bar{r}}_{s}}{z_{s1}}\right)Q\left(\frac{1}{z_{s1}}\right)\exp\left(-i2\pi \Lambda^{-1}R\right)⨂Q\left(\frac{1}{z_{s2}}\right)\right\}\exp\left\{-i\pi ({\lambda f)}^{-1}R^{2}\right\}$, and the intensity distribution at the image sensor located at a distance of *z_h_* from the lens is given as $I_{PSF}={\left|\left\{\sqrt{I_{s}}C_{1}L\left(\frac{{\bar{r}}_{s}}{z_{s1}}\right)Q\left(\frac{1}{z_{s1}}\right)\exp\left(-i2\pi \Lambda^{-1}R\right)⨂Q\left(\frac{1}{z_{s2}}\right)\right\}\exp\left\{-i\pi ({\lambda f)}^{-1}R^{2}\right\}⨂Q\left(\frac{1}{z_{h}}\right)\right|}^{2}.$ The intensity for an object *O* is given as $I_{O}=I_{PSF}⨂O$. For a single object point, the complex amplitude $\sqrt{I_{s}}C_{1}L\left(\frac{{\bar{r}}_{s}}{z_{s1}}\right)Q\left(\frac{1}{z_{s1}}\right)\exp\left(-i2\pi \Lambda^{-1}R\right)$ generates a magnified Bessel intensity distribution *J*_0_ at a distance of *z,* depending on the strength of the quadratic phase function. Now, the rest of the optical set up only reimages the Bessel distribution *J*_0_ with a transverse magnification given as *z_h_*/*z_s2_* and longitudinal magnification of (*z_h_*/*z_s2_*)^2^. This reimaging processing reorganizes the Bessel intensity distribution close to the lens with the far-field ring pattern at a larger distance. Depending upon the period Λ of the axicon, in the image plane, a Bessel distribution or a ring pattern is obtained for a point object in the object plane. Therefore, it is necessary to analyze the above equation and set the period Λ and distance *z_h_* such that the line focus of J_0_ is matched with the image plane of the system. It must be noted that, within the line focus, the magnification of image can vary depending upon the distances *z*_s1_, *z*_s2_, and *z_h_*. For instance, when a thin object is imaged, the object appears with different sizes within the image depth due to a one-many object-image mapping of the axicon instead of the usual one-one mapping in a lens.

Therefore, it is possible to obtain a Bessel beam even with a commercial microscope with intact objective lenses by introducing an axicon with a certain period Λ and at a certain distance. Based on spatially incoherent imaging, every object point is mapped to a Bessel intensity distribution, and there is intensity addition in the regions of overlap of Bessel beams generated for two different object points.

## 3. Simulation Studies

**Figure 2:**
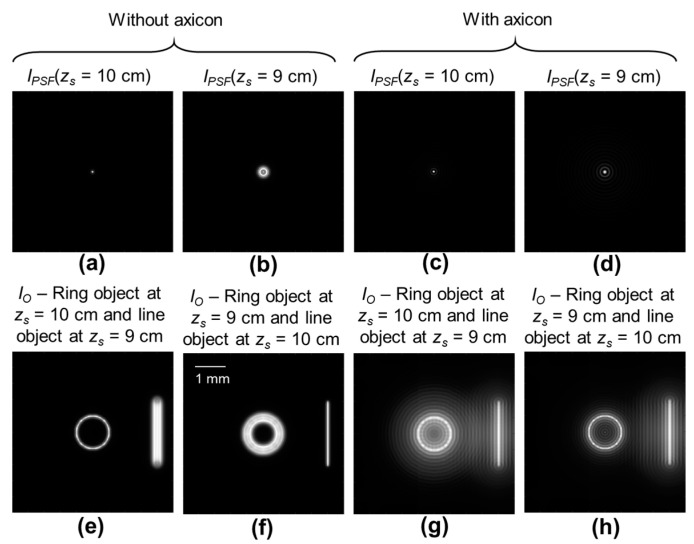
Simulated *I_PSF_* (**a**) *z_s_* = 1 m and (**b**) *z_s_* = 0.8 m for a regular microscope, and (**c**) *z_s_* = 1 m and (**d**) *z_s_* = 0.8 m for a microscope with an axicon. Simulated imaging outcomes for regular microscope when (**e**) ring object is in focus and (**f**) line object is in focus. Simulated imaging outcomes for microscope with an axicon when (**g**) ring object is in focus and (**h**) line object is in focus. The variable *z_s_* is the distance between the object and the lens.

A simulation study was carried out in MATLAB with a matrix size of 500 × 500 pixels, pixel size ∆ = 10 μm, and wavelength *λ* = 450 nm. For comparison between a regular microscope and the modified microscope with an axicon close to the object, the distances were selected as *z_s_* = *z_s_*_1_ + *z_s_*_2_ = 10 cm, where *z_s_*_1_ = 5 cm, *z_s_*_2_ = 5 cm, *z_h_* = 5 cm, and the focal length of the refractive lens is *f* = 3.5 cm. Now two objects, a ring object with a diameter of 1 mm and a line object with a length of 2 mm and both with a thickness of 10 μm, were simulated at distances *z_s_* = 10 cm and *z_s_* = 9 cm, respectively. The simulated *I_PSF_*s corresponding to the two locations *z_s_* = 10 cm and *z_s_* = 9 cm for a regular microscope and a microscope with an axicon are shown in Figure 2a–d, respectively. As expected, the *I_PSF_* for the case without axicon vary with changes in *z_s_*, whereas the *I_PSF_* for the case with an axicon do not vary as much, as the case without axicon. However, the other maxima seen around the central maxima as rings in Figure 2c,d cause background effects. The imaging results of the two-plane object using a regular microscope and microscope with an axicon when focusing on the two planes *z_s_* = 10 cm and *z_s_* = 9 cm are shown in Figure 2e–h, respectively. As it is seen from the results, with an axicon, the depth of focus of a regular microscope is improved, with an additional background noise arising due to the sidelobes of the Bessel distribution. The only drawback in the case of a microscope with an axicon is the loss or suppression of some higher spatial frequencies in the frequency domain due to the sidelobes of the Bessel function and additional background noise. Even though the simulation study has been demonstrated for visible wavelength, the results can be scaled to any wavelength without the loss of generality as the pixel size ∆ can be tuned to obtain a wavelength-independent behavior.

**Figure 3:**
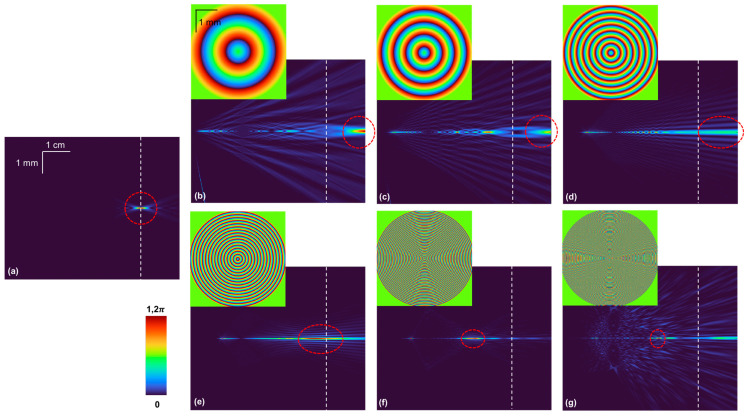
Images of axial distributions of *I_PSF_* along (*x*,*z*) for the optical configurations (**a**) without an axicon and with an axicon with a period Λ = (**b**) 800 μm, (**c**) 400 μm, (**d**) 200 μm, (**e**) 100 μm, (**f**) 50 μm, and (**g**) 25 μm. White dotted line indicates sensor location and red dotted circle indicates depth of focus region of the axicon.

The simulation study is extended for diffractive axicons with different periods Λ = 800 μm, 400 μm, 200 μm, 100 μm, 50 μm, and 25 μm, and the axial distribution is compared with that of the case without an axicon for the distances set in the simulation study. The axial distributions of intensity along (*x*,*z*) were obtained by simulating the *I_PSF_* using the equation, derived in the previous section, $I_{PSF}={\left|\left\{\sqrt{I_{s}}C_{1}L\left(\frac{{\bar{r}}_{s}}{z_{s1}}\right)Q\left(\frac{1}{z_{s1}}\right)\exp\left(-i2\pi \Lambda^{-1}R\right)⨂Q\left(\frac{1}{z_{s2}}\right)\right\}\exp\left\{-i\pi ({\lambda f)}^{-1}R^{2}\right\}⨂Q\left(\frac{1}{z_{h}}\right)\right|}^{2}$ for different values of *z_h_* from 0 to 5 cm. The images of the axial distributions along (*x*,*z*) for the case without an axicon is shown in Figure 3a. The images of the axial distributions for the cases with an axicon with different periods Λ = 800 μm, 400 μm, 200 μm, 100 μm, 50 μm, and 25 μm are shown in Figure 3b–g, respectively. The position of the sensor is indicated with a white dotted line and the regions of image formation are shown with a red dotted circle. As seen from the simulation results, the case without an axicon satisfies the imaging condition exactly at the plane of the sensor, and so a focused image is obtained for this case. For the other cases with axicon, the location of the line focus varies with the period of the axicon. Only for the cases in Figure 3d,e, the line focus matches well with the plane of the sensor. Therefore, in other cases, where the line focus region does not match with the sensor plane, it is necessary to shift the location of the sensor once. Therefore, for extending the depth of focus of microscopes without modifying its configuration, the Λ of the axicon and the location of the axicon must be input in the above equation to obtain the optimal Λ and location. Since there are two degrees of freedom, if one of two degrees is fixed, such as a prefabricated axicon, then the location can be adjusted. If there is limited clearance for changing the location, an axicon with a different period can be used.

## 4. Optical Experiments with a Solid Axicon

**Figure 4:**
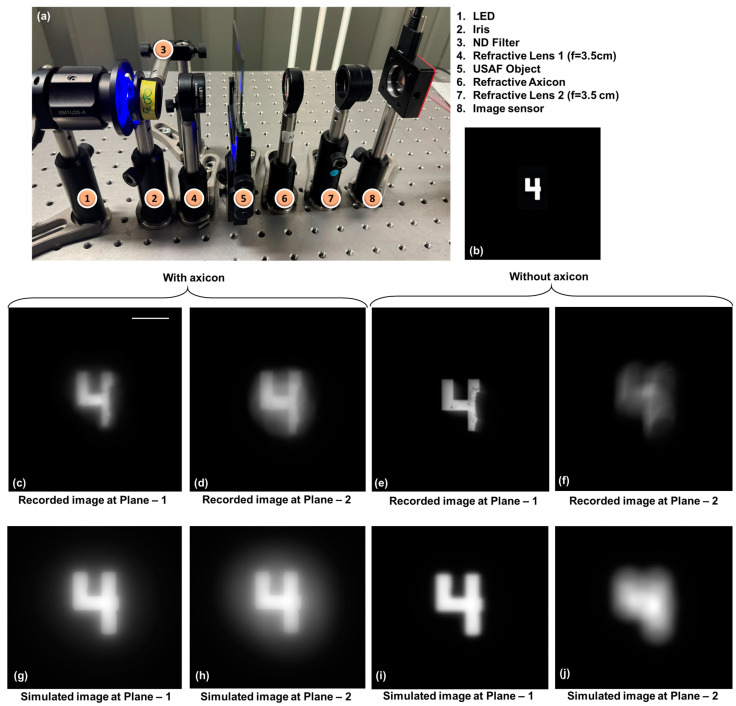
(**a**) Photograph of the experimental set up. (**b**) Test object. Experimental imaging results with axicon (**c**) plane 1 and (**d**) plane 2. Experimental imaging results without axicon for (**e**) plane 1 and (**f**) plane 2. Simulation results with axicon (**g**) plane 1 and (**h**) plane 2. Experimental imaging results without axicon for (**i**) plane 1 and (**j**) plane 2. Scale bar 0.5 mm.

An optical experiment was carried out using a solid axicon with a base angle of 2.5 degrees, a thickness of 1 cm, and a diameter of 2.5 cm with a refractive index of ~1.5 (632.8 nm) (Optek Systems, Riga, Latvia) and a refractive lens with a focal length *f* = 3.5 cm similar to the schematic of Figure 1b. The distance between the object and lens was 10 cm, and the distance between lens and sensor was 5 cm. The axicon was mounted close to the object by ~5 cm. The picture of the setup is shown in Figure 4a. The object digit ‘4’ (Group—0, Element—4) (Figure 4b) from USAF resolution target (Thorlabs, R3L3S1N negative target) was illuminated with a blue LED (λ = 450 nm and ∆λ = 18 nm) using a refractive lens. An ND filter was used to reduce the light intensity. The image was recorded using an image sensor (Zelux CS165MU/M 1.6 MP monochrome CMOS camera, 1440 × 1080 pixels with pixel size ~3.5 µm, Thorlabs, Newton, MA, USA). Two recordings were made at two different planes separated by a distance of 1 cm. Depending upon the base angle of the solid axicon or period of the diffractive axicon, the location of the image formation varied. With the current solid axicon, the image sensor was moved by ~2 cm. The images of the object recorded with an axicon at planes 1 and 2 are shown in Figure 4c and d, respectively. The images of the object recorded without an axicon at planes 1 and 2 are shown in Figure 4e and f, respectively. In the cases of recordings in the presence of an axicon, the object information at both planes was simultaneously focused with additional background noise. In the two cases without an axicon, the object information when the imaging condition was satisfied appeared focused, whereas the object information when the imaging condition was disturbed by 1 cm appeared blurred, as expected. There are three effects when an axicon is introduced into an imaging system. (1) Within the focal depth of the axicon, the image appears focused but can be magnified depending upon the distance. (2) The sidelobes of the Bessel function suppresses some of the higher spatial frequencies in the Fourier domain, resulting in a slight blur in the recorded images. (3) The other non-central intensity maxima of the Bessel distribution cause a background effect. A simulation study was carried out using the same test object by inputting it into the software as an image but with a diffractive axicon and a single wavelength matching the specifications of the experiment. The images of the object with an axicon at the two planes separated by a distance of 1 cm are shown in Figure 4g and h, respectively. The images of the object without an axicon at the two planes separated by a distance of 1 cm are shown in Figure 4i and j, respectively. A good overlap between the simulation and the experiments is observed.

## 5. Optical Experiments with a Spatial Light Modulator

**Figure 5:**
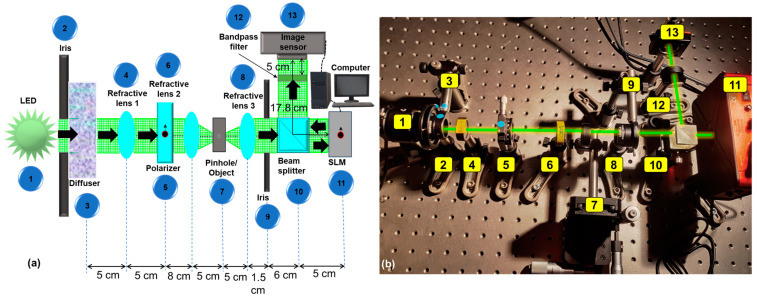
(**a**) Schematic of the experimental set up. (**b**) Snapshot of optical experimental setup: (1) high-power green LED, (2) iris, (3) diffuser, (4) refractive lens 1, (5) polarizer, (6) refractive lens 2, (7) object, (8) refractive lens 3, (9) iris, (10) beam splitter, (11) SLM, (12) band pass filter, and (13) image sensor. The green line shows the beam path from LED to image sensor.

**Figure 6:**
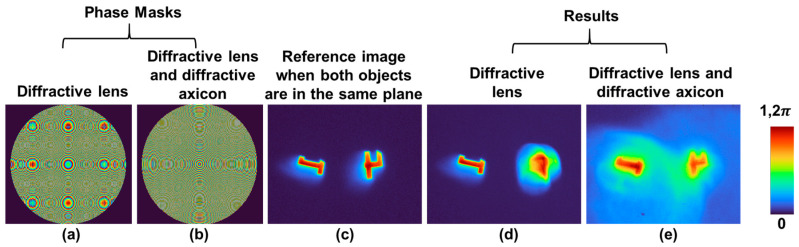
Images of phase masks of (**a**) diffractive lens and (**b**) combination of diffractive axicon and diffractive lens. (**c**) Image recorded with both objects digit ‘1’ and ‘4’ in the same plane. Images (**d**) and (**e**) recorded with (**a**) and (**b**), respectively.

A modified optical experiment of multiplane imaging was carried out using an SLM in a setup whose schematic and photograph are shown in Figure 5a and b, respectively. In this study, the phase of the diffractive axicon was combined with the phase of a diffractive lens, and the imaging was carried out in the near field. The setup consisted of high-power green LED (Thorlabs MINTL5, λ = 554 nm and 650 mW), an iris, a diffuser (Thorlabs Ø1” Ground Glass Diffuser-220 GRIT), a polarizer, a refractive lens (f = 5 cm), an object (Group 3—digits ‘1’ and ‘4’ from Thorlabs R1DS1N-Negative 1951 USAF Test Target), a beam splitter, an SLM (Thorlabs Exulus HD2, 1920 × 1200 pixels, pixel size = 8 μm), a band pass filter (Thorlabs FLH532-4, λ = 532 nm and Δλ = 4 nm), and an image sensor (Zelux CS165MU/M 1.6 MP monochrome CMOS camera, 1440 × 1080 pixels with pixel size ~3.5 µm). The light from the LED entered the diffuser through an iris and was collected using the refractive lens 1. The diffuser was used here to remove the grating lines and other noises from the LED. The collimated light from the refractive lens 1 entered the polarizer oriented along the active axis of the SLM. The object was critically illuminated using the refractive lens 2, and the light from the object was collimated using refractive lens 3. The collimated light entered the beam splitter and reached the SLM, illuminating its entire area. On the SLM, phase masks were displayed, and the images were captured by the image sensor placed at a distance *z_h_* = 17.8 cm. A bandpass filter was used to improve the visibility of the recordings. The test objects, namely digits ‘1’ and ‘4’, were mounted at different depths (5 cm and 5.1 cm, respectively) with respect to the refractive lens 3 and recorded one after another and summed. Since the illumination source was a spatially incoherent one, the light diffracted from the two points did not interfere with one another, but their intensities added up. Therefore, the above approach is identical to a two-channel optical experiment. The phase mask of a diffractive lens (*f* = 17.8 cm) and the phase mask obtained by a modulo-2π phase addition of the phase of a diffractive lens (*f* = 50 cm) and a diffractive axicon (Λ = 200 μm) are shown in Figure 6a and b, respectively. The direct image, when both objects are at the same depth, is shown in Figure 6c. The optical imaging results for using the diffractive lens only and the combination of a diffractive lens with the diffractive axicon are shown in Figure 6d and e, respectively. In cases with a diffractive lens, the object ‘1’ is focused, and object ‘4’ is blurred. But in the cases with a diffractive lens-diffractive axicon, both objects ‘1’ and ‘4’ are in focus. However, there is an additional background noise arising due to the nature of the Bessel distribution with a central maxima and less intense successive maxima in the form of multiple ring patterns around the central maxima.

## 6. Fabrication of Binary Axicon

Two binary axicons were manufactured on barium fluoride (BaF_2_) substrates (Crystran, Poole, UK) with a diameter of an inch and thickness of 1 mm using two different methods, namely photolithography and femtosecond ablation as an amplitude and phase element, respectively. The maximum diffraction efficiencies for amplitude and binary phase elements are 10% and 40%, respectively, in the first diffraction order [39,40]. The BaF_2_ substrates, unlike glass substrates, are brittle and can be easily damaged. The design for photolithography was made in MATLAB (Version R2022a), and the image file was converted into lithography file format GDSII using LinkCAD software (Trial version). To limit the memory of the file size, the sampling size was set to a low value, which resulted in pixelated rings.

**Figure 7:**
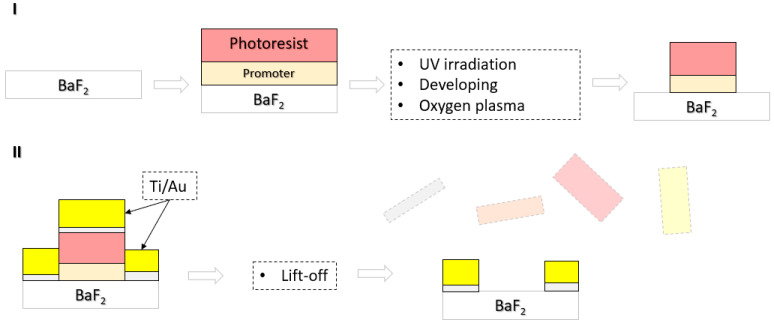
Fabrication procedure of binary axicon using photolithography. BaF_2_—barium fluoride. Ti and Au stands for titanium and gold, respectively. (**I**)—Photolithography (coating photoresist, UV irradiation and developing). (**II**)—Metal evaporation and removal of excess metal and photoresist.

The first binary axicon was manufactured with a period Λ = 100 μm using a photolithography technique in an ISO5 clean-room. The entire fabrication procedure in two major steps is shown in Figure 7. The positive photoresist (AR-P 3510T, Allresist, Strausberg, Germany) was spin coated (4000 rpm, 60 s) with a thickness of 2 µm onto BaF_2_ substrates and then softly baked on a hot plate at 100 °C for 60 s. The promoter AR 300-80 (15–30 nm) (Allresist, Germany) was used to improve the adhesion between the photoresist and the BaF_2_ substrates. Maskless Aligner (Heidelberg Instruments µMLA, Heidelberg, Germany) with a dose control of the light source at 390 nm was used to expose the photoresist, AR 300-44 (Allresist, Germany) was used for developing the UV irradiated structures, and ultrapure water was used to remove possible residuals.

**Figure 8:**
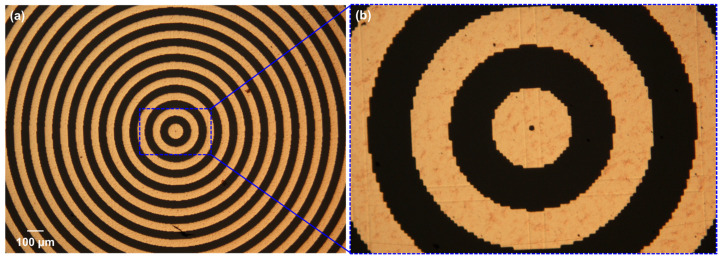
(**a**) Optical microscope image of the binary axicon and (**b**) its magnified central part manufactured using photolithography.

Before metallization, the surface of BaF_2_ was treated with oxygen plasma. Thereafter, Ti and Au (the thicknesses approximately 6 nm and 100 nm, respectively) was electron-beam evaporated at room temperature in a process vacuum of about 2 × 10^−6^ mbar with a growth rate of ~2 Å/s. A thin Ti layer was used to improve the adhesion between Au and the glass. Finally, the lift-off procedure was carried out in an ultrasonic bath filled with warm acetone to remove excess metal and photoresist. The optical microscope image of the binary axicon and the magnified central area are shown in Figure 8a and b, respectively. The magnified central part of the device seen in Figure 8b shows the pixelated effects due to the limited resolution of the design file.

**Figure 9:**
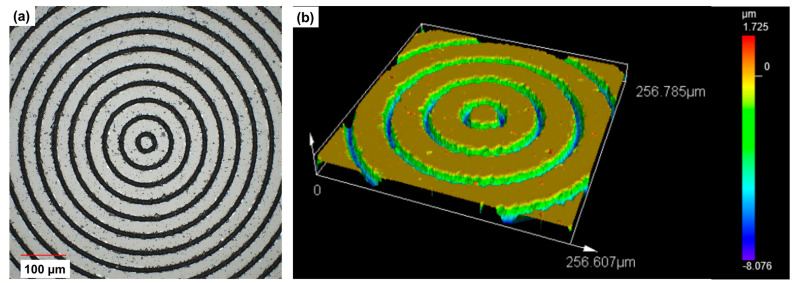
(**a**) Optical microscope image of the binary axicon and (**b**) surface profile of the central region (256.6 μm × 256.7 μm) of the binary axicon fabricated using femtosecond ablation.

The second binary axicon was manufactured using femtosecond ablation with a period Λ = 50 μm. The sample was fabricated using a femtosecond pulsed direct laser write approach. The fundamental harmonic (1030 nm) of an ultrashort pulsed solid-state Yb:KGW laser system (Pharos, Light Conversion, Vilnius, Lithuania) was directed to an external harmonic generator (HIRO, Light Conversion), where the beam was converted to a 4th harmonic (257 nm). The laser was working at a repetition rate of 100 kHz, generating 240 fs duration pulses. The pulse energy was set to 1.21 μJ, and the beam was focused on a sample using a Thorlabs LMU-10X-UVB objective with NA = 0.24. The incident beam diameter (at 1/e^2^ intensity level) was measured using a CCD camera (Spiricon SP503U, Ophir, Darmstadt, Germany) and was 2\ω_0_ = 4.6 mm. The sample was scanned on precise translation stages (Aerotech ANT 180, Pittsburgh, PA, USA) at a linear velocity of 500 μm/s. The optical microscope image of the binary axicon is shown in Figure 9a. The measured 3D surface topography is shown in Figure 9b, and the extracted 2D profiles reveal a depth of ~6.4 μm. This value gives an expected maximum efficiency of 40% at λ = ~5 μm with a refractive index of BaF_2_ is 1.4. The fabrication duration for a single device was 5 h. It must be noted that the above two fabrication techniques were used as they were available, and there are different fabrication methods for manufacturing a binary axicon which can be used [41,42,43].

## 7. Experiments with Synchrotron IR Beam

**Figure 10:**
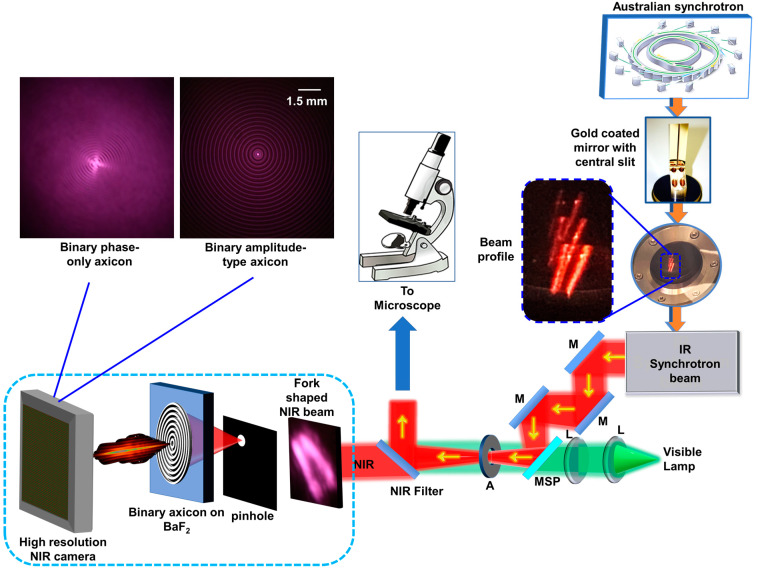
Optical configuration for testing the amplitude-type and phase-only binary axicons with synchrotron NIR beam. BaF_2_—barium fluoride; A—aperture; NIR—near infrared; L—lens; M—mirror; and MSP—motorized sliding plate.

The fabricated devices were tested with the synchrotron IR beam using an optical configuration, as shown in Figure 10, similar to a recently demonstrated phase imaging concept [44]. The IRM beamline at the Australian Synchrotron consists of two IR microscopes, one with Globar™ IR source and one with synchrotron IR beam with a high brightness. The IR synchrotron beam has a broad spectral range from 0.5 μm to 100 μm [45]. The NIR used in the experiment was between 800 nm and 2500 nm. In particular, the fabricated binary axicons were tested with a synchrotron NIR beam using a high-resolution NIR sensitive CMOS camera Canon EOS 6D (5568 × 3708 pixels) with a pixel pitch of 6.5 μm. The camera was used without the imaging lenses attached. The synchrotron NIR beam was extracted using a NIR window, which blocks all the IR wavelengths except for those in the NIR spectral range.

The synchrotron IR beam has a unique fork-shaped intensity distribution, which makes implementing unconventional imaging techniques such as FINCH and coded-aperture imaging a challenging task [10,46]. A pinhole with a diameter of 200 μm was mounted in the path of the beam, and its position was adjusted along the transverse direction until it was aligned with the maxima of the fork-shaped beam. The pinhole extracted a uniform circular region from the fork-shaped beam, which underwent diffraction, and an Airy diffraction pattern was obtained. The binary axicons were mounted in this plane of Airy centrosymmetric pattern, and the intensity distribution of the Bessel beam was recorded at a distance of ~10 cm. The images of the Bessel distributions obtained for the amplitude-type binary axicon and phase-only binary axicon are shown in Figure 10. The Bessel distributions generated by the two axicons were recorded under similar exposure conditions. As seen from the intensity distribution, the Bessel distribution from the binary phase axicon is stronger than that from the binary amplitude axicon, indicating a higher diffraction efficiency.

## 8. Experiments with IR Microscope

**Figure 11:**
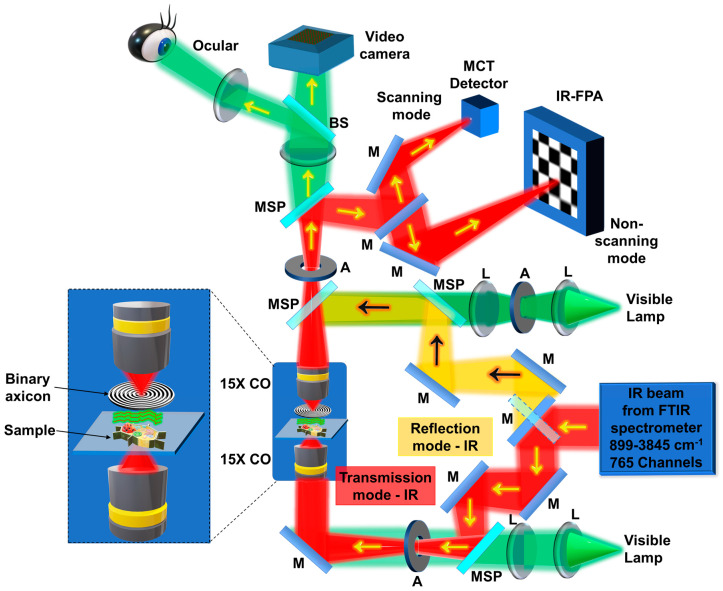
Schematic of the IR microscope. CO—Cassegrain objective; M—mirror; L—lens; IR—infrared; BS—beam splitter; FPA—focal point array; MSP—motorized sliding plate; FTIR—Fourier transform infrared spectrometer; MCT—mercury–cadmium–telluride; A—aperture; PSF—point spread function. The MIR from the FTIR spectrometer is sent into the IR/VISIBLE microscope. The microscope is aligned collinearly for both MIR and reference visible light. There are two modes of operation, reflection and transmission, and two modes of recording using MCT detector with scanning and FPA without scanning, respectively.

**Figure 12:**
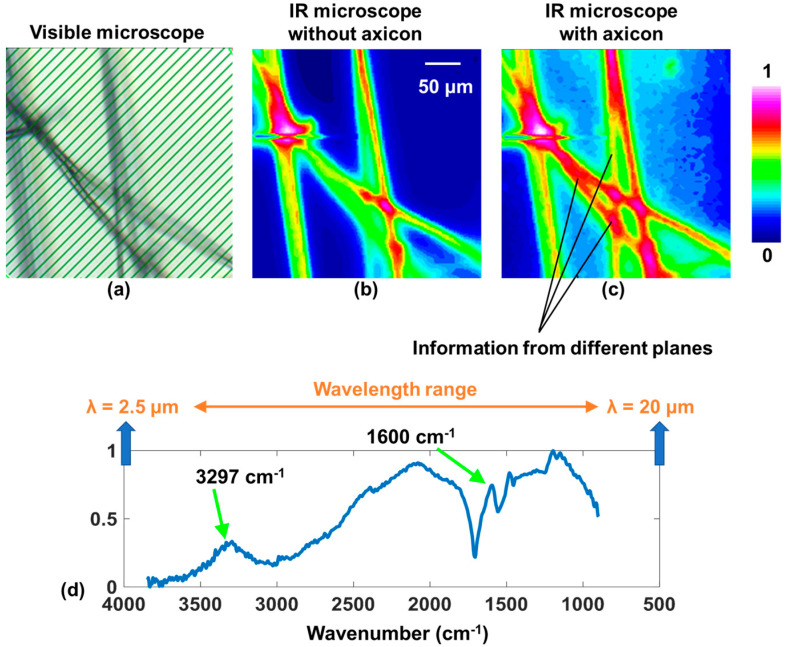
(**a**) Reference visible image of silk sample with white light. (**b**) Chemical images of silk sample taken without and (**c**) with a binary axicon. (**d**) Normalized IR absorption spectrum of silk sample. IR—infrared; λ—wavelength.

The schematic of the IR microscope is shown in Figure 11. Light from the Globar^TM^ source enters the spectrometer, and, from the spectrometer, it enters the IR microscope. Depending upon the operating mode, reflection or transmission, the orientation of the mirror M at the entrance is adjusted. In this study, the IR microscope was operated in the transmission mode. The IR light after the reflections from the mirrors was filtered by an aperture, collimated, and entered the 15× Cassegrain objective (CO), which critically illuminated the sample. The light from the sample was collected using a second 15× CO and imaged on a liquid nitrogen-cooled 64 × 64 element focal point array (FPA) mercury–cadmium–telluride (MCT) detector with a pixel size of 40 μm. The IR microscope can also be operated with a single pixel MCT detector and scanning mode for imaging with a higher field of view and resolution. In this research, the recordings were made using an FPA in non-scanning mode. To demonstrate imaging of multiple planes, a thick but sparse sample, such as a bunch of silk fiber, was selected and mounted on the standard sample mounting plane. Two cases were recorded and compared, including one without and one with the binary axicon. The binary axicon was mounted close to the sample, as shown in Figure 1 and Figure 11. For the final demonstration, only the phase-only binary axicon was selected due to the low power illumination from the internal Globar™ IR source. However, if the IR source had a high brightness, both phase-only and amplitude-type binary axicons could be used. The images were captured using a liquid nitrogen cooled 64 × 64 element focal point array (FPA) mercury–cadmium–telluride detector. The image capturing and processing were controlled using OPUS v.8.0 software (Bruker Optik GmbH, Ettlingen, Germany). Figure 12a shows the visible image of the silk sample that was first recorded using the visible camera equipped in the IR microscope. Two IR chemical images were subsequently collected using the FPA imaging detector on the same location with and without an axicon, as presented in Figure 12b and c, respectively. The images were obtained by selecting the narrow absorption band of the silk sample, shown in Figure 12c, followed by integration of the information in that spectral region. By comparison, the results clearly reveal an improved focal depth in the image taken with the axicon compared to without the axicon. The IR absorption spectrum of the silk sample is shown in Figure 12d with characteristic IR peaks of the silk within the MIR spectral region. It is worth noting that there is additional background noise and a slightly lower resolution when imaging with an axicon due to the sidelobes and non-central intensity maxima of the Bessel distribution. The sample was illuminated using a broadband IR region 899–3845 cm^−1^, which corresponds to ~2.5–12 μm. As seen from the characterization results in Figure 8 and the diffraction’s efficiency dependency on thickness, the optimal efficiency of 40% can be obtained at ~5 μm, and the efficiency decreases at other wavelengths [39,40]. The absorption peak of the silk sample was around 6.2 μm and, therefore, had a reasonable efficiency of ~35%.

## 9. Conclusions and Future Perspectives

Extending the focal depth of imaging systems using axicons is currently a tested method, but numerous configurations have been demonstrated in the past for different applications. In this study, we present a non-invasive approach to extend the focal depth of an IR microscope using binary axicons manufactured on BaF_2_ substrates. Simulation and experimental studies with both visible and IR wavelengths have been carried out.

The idea is that, when axicons are used with lenses, there are both spherical and conical phases in play [47,48,49,50,51,52,53]. Depending upon the distances and configurations, it is possible to access the near or far fields. The spherical phase either compresses or stretches the Bessel distribution of the conical phase axially depending on the cumulative effects of the phases of the lenses and propagation distances. In the near field, a Bessel distribution is obtained, which is the region of interest for extending the focal depth. In the far-field, for every object point, a focused ring pattern is generated, where the focusing effect can be attributed to the spherical phase. Multiple optical experiments with visible light have been demonstrated.

The final experiment has been carried out in the IR microscope of the Australian Synchrotron with an internal Globar™ IR source. The preliminary results are promising, as multiple planes of the test object have been successfully imaged in a single shot. We believe that the developed method and device will benefit the ongoing research and development for the users of the Australian Synchrotron’s IR beamline, as well as other synchrotron IR facilities. In the future, greyscale axicon with a high diffraction efficiency will be manufactured on BaF_2_. Research on suppressing the sidelobes and background and achieving needle beams will enable the imaging of thicker samples with sparse distributions rapidly.

## Data Availability

Data are available from the authors upon reasonable request.
